# Supplementary material for: Network Pharmacology and Molecular Docking-Based Approach to Explore Potential Bioactive Compounds from Kaempferia parviflora on Chemokine Signaling Pathways in the Treatment of Psoriasis Disease
Source: Int J Mol Sci. 2025 May 29;26(11):5243. doi: 10.3390/ijms26115243 (PMC12154073; doi:10.3390/ijms26115243)
Supplement: Supplementary file 1 [file ijms-26-05243-s001.zip › ijms-3630289-supplementary/Supplementary data Figures_ijms-3630289.pdf]

International Journal of
Molecular Sciences

MDPI

Supplementary Data

Network pharmacology and molecular docking-based approach to explore potential bioactive compounds from *Kaempferia parviflora* on chemokine signaling pathways in the treatment of psoriasis disease

Figure S1: Molecular docking of complex structure between AKT1-L8, AKT1-L11, and PIK3R1-L12

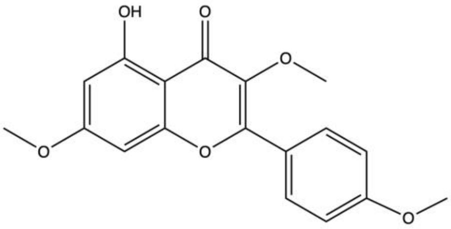

5-hydroxy-3,7,4'-trimethoxyflavone

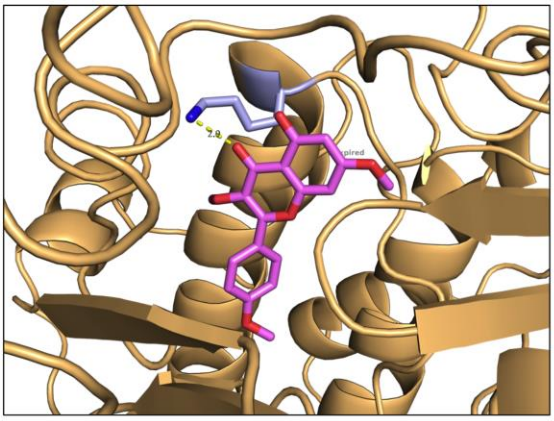

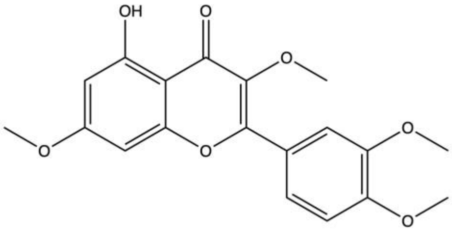

5-hydroxy-3,7,3',4'-tetramethoxyflavone

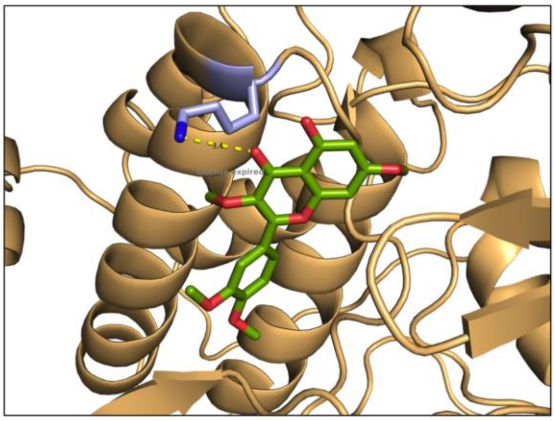

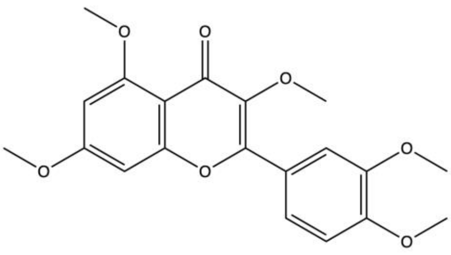

3,5,7,3',4'-pentamethoxyflavone

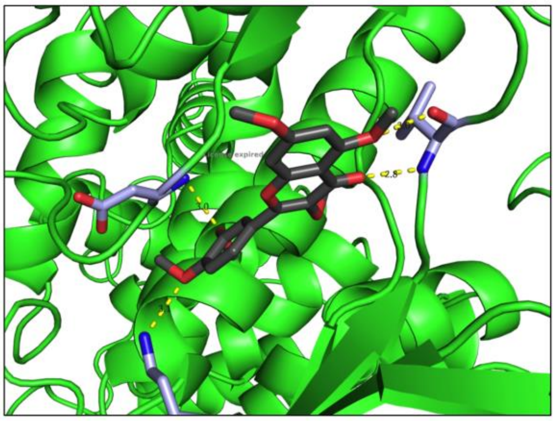

Figure S2: 2D Molecular Docking Analysis of Selected Compounds with SRC, AKT1, and PIK3R1.

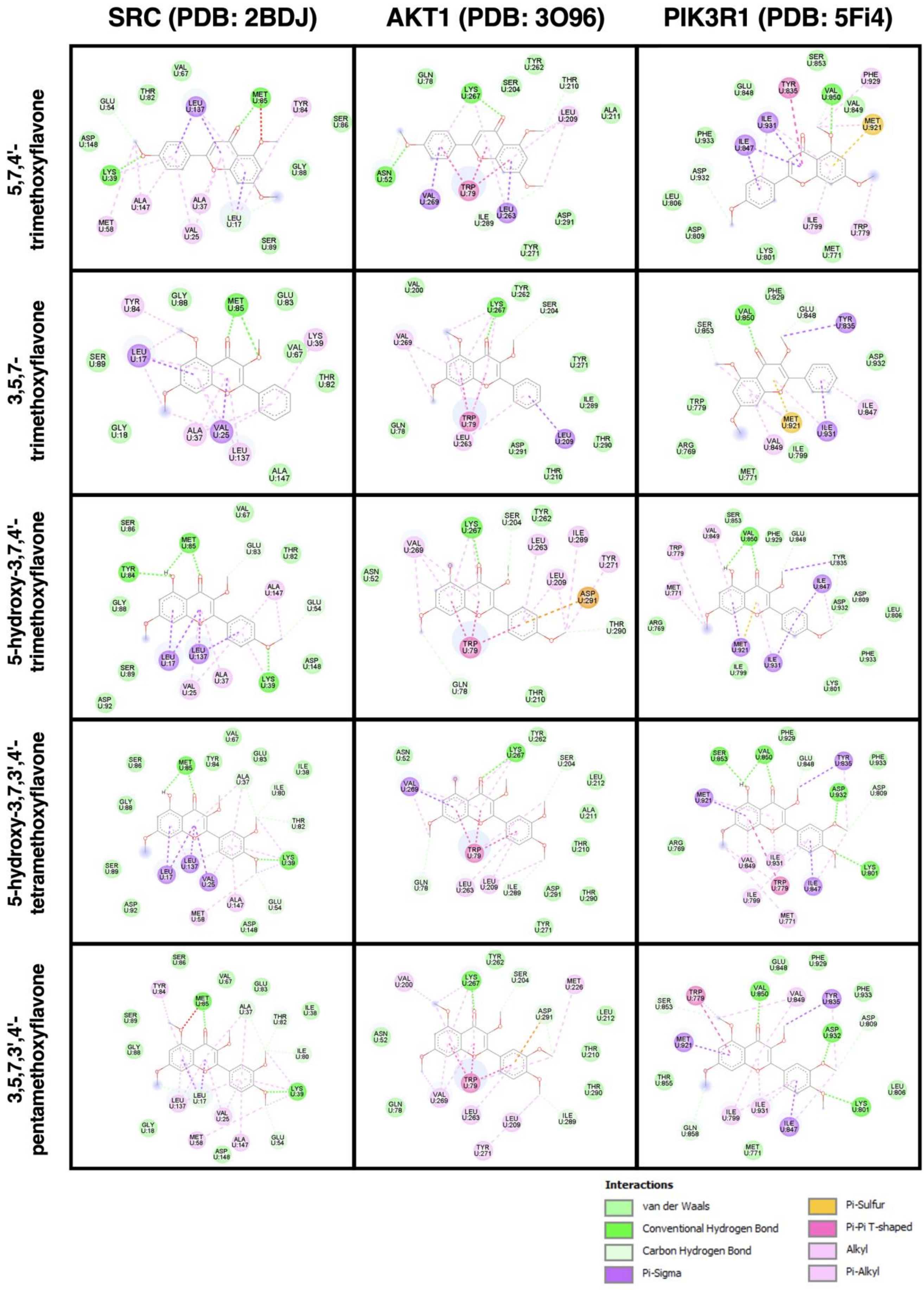

Figure S3: Chemical Structures of Methoxyflavones Isolated from KP

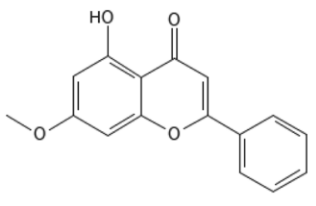

5-hydroxy-7-methoxyflavone

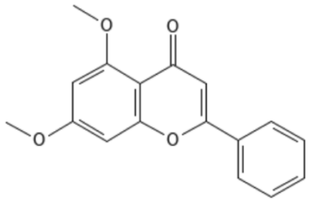

5,7-Dimethoxyflavone

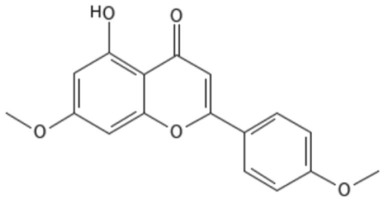

5-hydroxy-7,4`-dimethoxyflavone

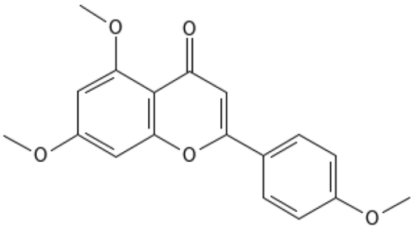

5,7,4' -trimethoxyflavone

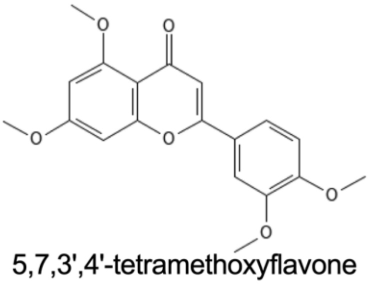

5,7,3',4'-tetramethoxyflavone

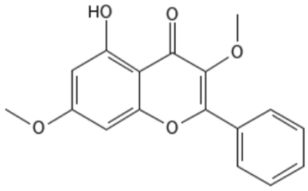

5-Hydroxy-3,7- dimethoxyflavone

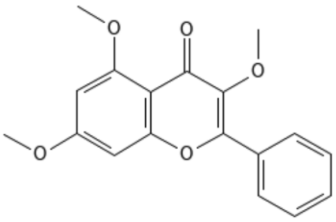

3,5,7-Trimethoxyflavone

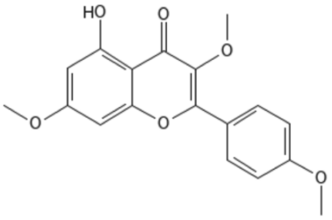

5-hydroxy-3,7,4`-trimethoxyflavone

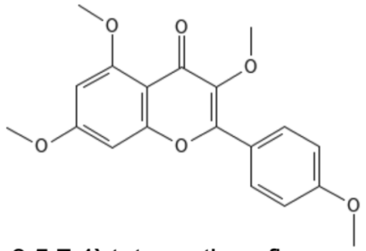

3,5,7,4`-tetramethoxyflavone

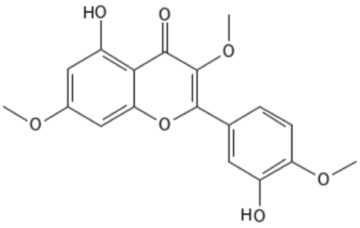

5,3'-dihydroxy-3,7,4'
-trimethoxyflavone

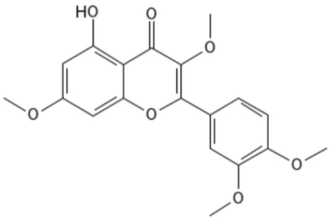

5-hydroxy-3,7,3`,4`-
tetramethoyflavone

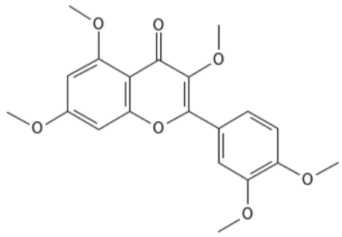

3,5,7,3', 4'- pentamethoxyflavone

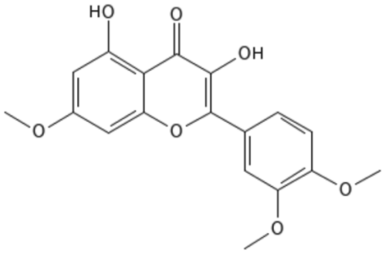

3,5-dihydroxy-7, 3',4'-trimethoxyflavone

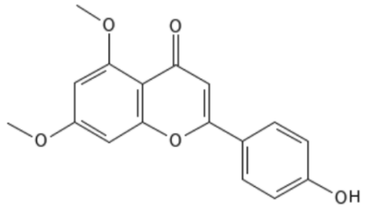

5,4'-dihydroxy-7-methoxyflavone

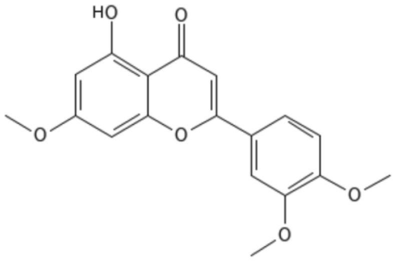

5-hydroxy-7, 3',4' -trimethoxyflavone

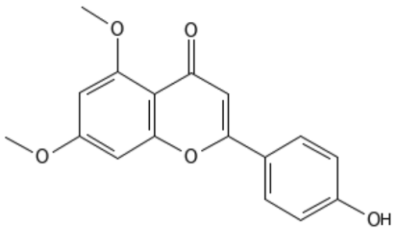

4'- hydroxy-5,7-dimethoxyflavone

Figure S4: Effects of Bioactive Compounds in KP on Nitric Oxide Production in LPS-Induced RAW264.7 Cells.

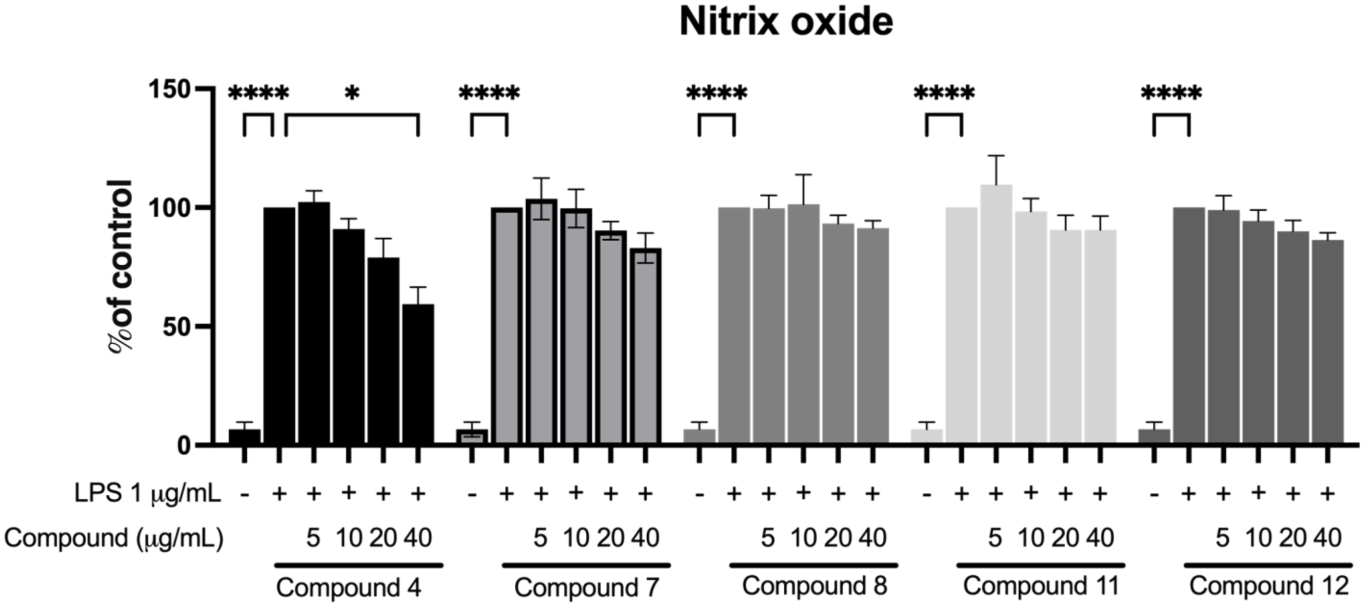

RAW264.7 macrophages were treated with LPS (1 μg/mL) to induce an inflammatory response, followed by exposure to different concentrations (0, 5, 10, 20, and 40 μg/mL) of each compound for 48 hours. Data are presented as mean ± S.D. from at least three independent experiments. Statistical significance was determined using one-way ANOVA followed by post hoc analysis (*$p < 0.05$ and ****$p < 0.01$). compound 4; 5,7,4'-trimethoxyflavone, compound 7; 3,5,7-trimethoxyflavone, compound 8; 5-hydroxy-3,7,4'-trimethoxyflavone, compound 11; 5-hydroxy-3,3',4',7-tetramethoxyflavone, and compound 12; 3,5,7,3',4'-pentamethoxyflavone.
