# Supplementary material for: Network Pharmacology and Molecular Docking-Based Approach to Explore Potential Bioactive Compounds from Kaempferia parviflora on Chemokine Signaling Pathways in the Treatment of Psoriasis Disease
Source: Int J Mol Sci. 2025 May 29;26(11):5243. doi: 10.3390/ijms26115243 (PMC12154073; doi:10.3390/ijms26115243)
Supplement: Supplementary file 1 [file ijms-26-05243-s001.zip › ijms-3630289-supplementary/TableS1.pdf]

Supplementary table S1. Toxicity parameters and pharmacokinetic properties of active ingredients in KP

No.	Compounds	Carcinogenicity	Mutagenicity	Hepatotoxicity	Rat oral acute toxicity	OB%	Caco-2	BBB	DL
1	5-hydroxy-7-methoxyflavone	--	+	---	---	9.57	0.97	0.27	0.20
2	5,7-Dimethoxyflavone	--	++	--	---	-	-	-	-
3	5-hydroxy-7,4`-dimethoxyflavone	--	+	--	---	27.12	0.83	0.03	0.27
4	5,7,4' -trimethoxyflavone	--	+	--	---	39.83	1.01	0.12	0.30
5	5,7,3',4'-tetramethoxyflavone	---	-	--	---	43.68	0.96	0.09	0.37
6	5-Hydroxy-3,7- dimethoxyflavone	--	+	--	---	-	-	-	-
7	3,5,7-Trimethoxyflavone	--	+	--	---	-	-	-	-
8	5-hydroxy-3,7,4`-trimethoxyflavone	---	+	--	---	-	-	-	-
9	3,5,7,4`-tetramethoxyflavone	---	+	--	---	-	-	-	-
10	5,3'-dihydroxy-3,7,4' -trimethoxyflavone	---	+	--	--	14.96	0.76	-0.29	0.37
11	5-hydroxy-3,7,3`,4`-tetramethoyflavone	---	-	--	--	25.45	0.79	0.05	0.40
12	3,5,7,3', 4'- pentamethoxyflavone	---	-	--	--	-	-	-	-
13	3,5-dihydroxy-7, 3',4'-trimethoxyflavone	---	+	--	--	-	-	-	-
14	5,4'-dihydroxy-7-methoxyflavone	--	+	---	---	37.13	0.63	-0.24	0.24
15	5-hydroxy-7, 3',4' -trimethoxyflavone	---	-	--	---	-	-	-	-
16	4'- hydroxy-5,7-dimethoxyflavone	--	+	--	---	-	-	-	-

TCMSP: Carcinogenicity: Indicates whether the compound has potential cancer-causing effects; Mutagenicity: Assesses whether the compound can cause genetic mutations; Hepatotoxicity: Evaluates the potential toxic effects on the liver; Rat oral acute toxicity: Measures the immediate toxic effects when administered orally to rats; OB% (Oral Bioavailability Percentage): Represents the fraction of an orally administered drug that reaches systemic circulation. Higher values suggest better absorption; Caco-2: Refers to permeability across Caco-2 cells, a model for predicting intestinal absorption in humans; BBB (Blood-Brain Barrier Penetration): Predicts the ability of the compound to cross the blood-brain barrier, with positive values suggesting potential CNS activity; DL (Drug-Likeness Score): A computational measure predicting the compound's likelihood of being an orally active drug based on its physicochemical properties.
