# Supplementary material for: Network Pharmacology and Molecular Docking-Based Approach to Explore Potential Bioactive Compounds from Kaempferia parviflora on Chemokine Signaling Pathways in the Treatment of Psoriasis Disease
Source: Int J Mol Sci. 2025 May 29;26(11):5243. doi: 10.3390/ijms26115243 (PMC12154073; doi:10.3390/ijms26115243)
Supplement: Supplementary file 1 [file ijms-26-05243-s001.zip › ijms-3630289-supplementary/TableS6.pdf]

Supplementary table S6. A structured table clarifying the atom numbering positions for the 16 methoxyflavones from *Kaempferia parviflora* (KP), based on the standard flavonoid numbering system.

Compound Name	Methoxy Substitution Positions
5,7-Dimethoxyflavone	C5, C7 (A-ring)
3,5,7-Trimethoxyflavone	C3, C5, C7 (A-ring)
5,7,4'-Trimethoxyflavone	C5, C7 (A-ring), C4' (B-ring)
3,5,7,3',4'-Pentamethoxyflavone	C3, C5, C7 (A-ring), C3', C4' (B-ring)
5-Hydroxy-7-methoxyflavone	C7 (A-ring)
5-Methoxyflavone	C5 (A-ring)
7-Methoxyflavone	C7 (A-ring)
5,7-Dimethoxy-4'-hydroxyflavone	C5, C7 (A-ring)
3,5-Dimethoxyflavone	C3, C5 (A-ring)
5,7,3',4'-Tetramethoxyflavone	C5, C7 (A-ring), C3', C4' (B-ring)
5,7,8-Trimethoxyflavone	C5, C7, C8 (A-ring)
5,6,7-Trimethoxyflavone	C5, C6, C7 (A-ring)
5,7-Dimethoxy-3'-hydroxyflavone	C5, C7 (A-ring)
5,7,3',5'-Tetramethoxyflavone	C5, C7 (A-ring), C3', C5' (B-ring)
5,7,4'-Trimethoxy-3-hydroxyflavone	C5, C7 (A-ring), C4' (B-ring)
5,7,2',4'-Tetramethoxyflavone	C5, C7 (A-ring), C2', C4' (B-ring)
