# Supplementary figures and images for: Network Pharmacology and Molecular Docking-Based Approach to Explore Potential Bioactive Compounds from Kaempferia parviflora on Chemokine Signaling Pathways in the Treatment of Psoriasis Disease
Source: Int J Mol Sci. 2025 May 29;26(11):5243. doi: 10.3390/ijms26115243 (PMC12154073; doi:10.3390/ijms26115243)

5,7,4'-trimethoxyflavone (C1) - Figure 7A

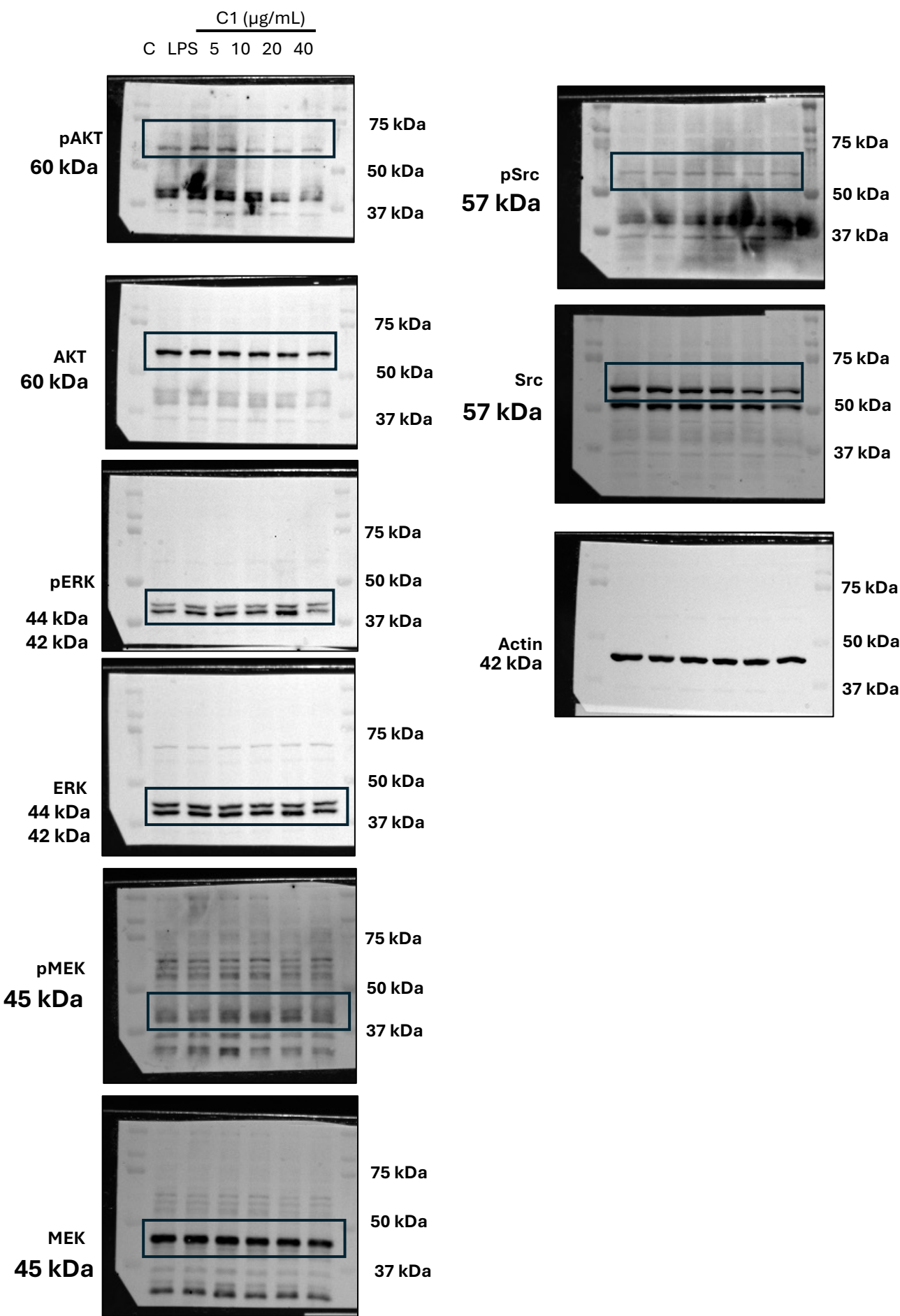

3,5,7-trimethoxyflavone (C2) – Figure 7B

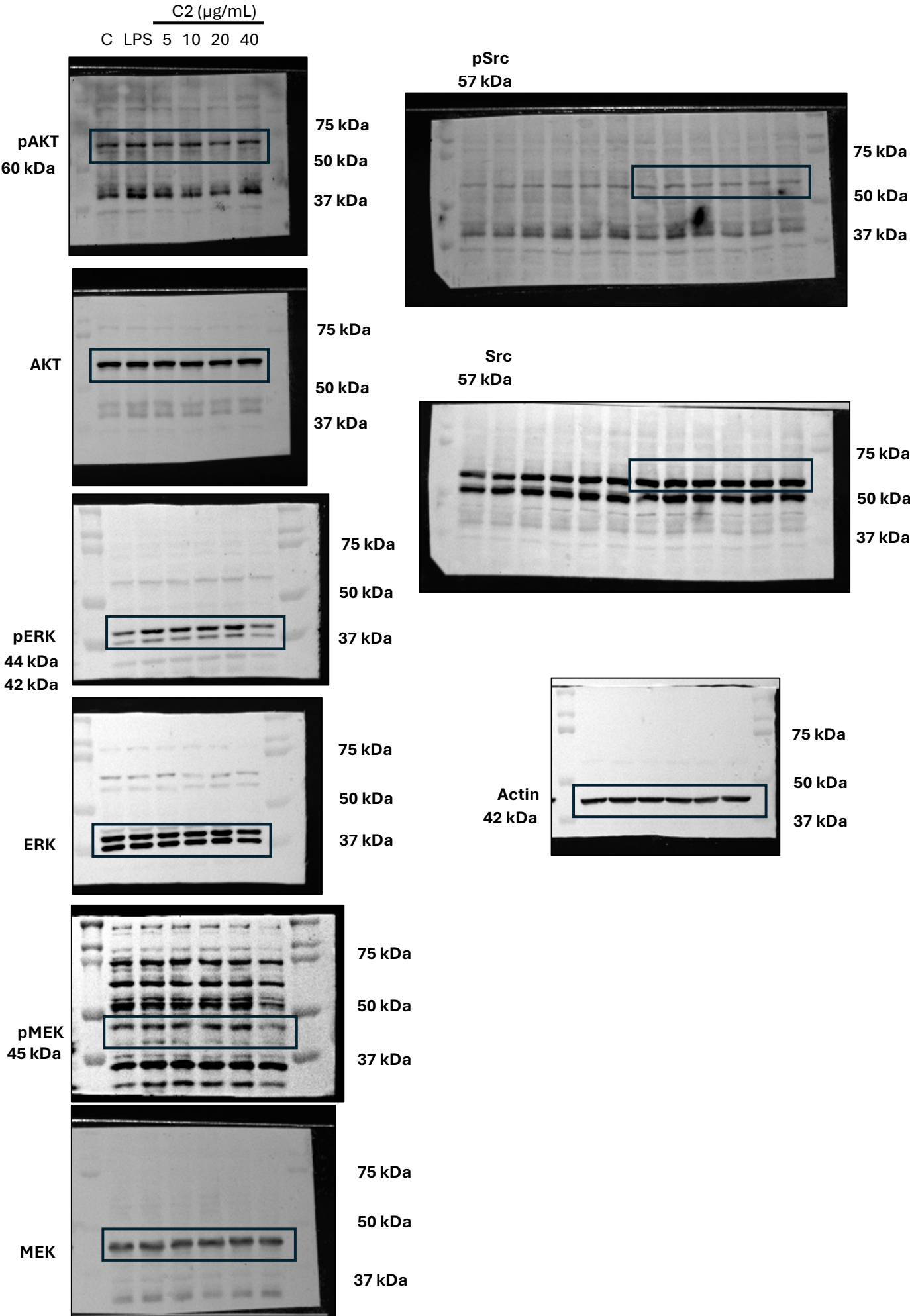

Supplement: Supplementary file 1 [file ijms-26-05243-s001.zip › ijms-3630289-supplementary/Original blot (Figure 8).pdf]
